# Dual time point imaging in locally advanced head and neck cancer to assess residual nodal disease after chemoradiotherapy

**DOI:** 10.1186/s13550-022-00905-y

**Published:** 2022-06-13

**Authors:** Frederik Soffers, Nils Helsen, Tim Van den Wyngaert, Laurens Carp, Otto S. Hoekstra, Laurence Goethals, Michel Martens, Kristof Deben, Karoline Spaepen, Remco De Bree, Frank De Geeter, G. J. C. Zwezerijnen, Carl Van Laer, Alex Maes, Olivier Lenssen, Sigrid Stroobants, the ECLYPS Investigators, Laurence Beels, Jean-Philippe Cambier, Laurens Carp, Kristof Deben, Remco De Bree, Philip Debruyne, Frank De Geeter, Laurence Goethals, Sara Hakim, Nils Helsen, Otto S. Hoekstra, Filip Homans, Isabel Hutsebaut, Olivier Lenssen, Alex Maes, Annelies Maes, Michel Martens, Karoline Spaepen, Pol Specenier, Sigrid Stroobants, Daniëlle van den Weyngaert, Tim Van den Weyngaert, Olivier Vanderveken, Joost van Dinther, Carl Van Laer, G.J.C. Zwezerijnen

**Affiliations:** 1 Department of Nuclear Medicine, Antwerp University Hospital, Edegem, Belgium; 2 Faculty of Medicine and Health Sciences, University of Antwerp, Antwerp, Belgium; 3 Faculty of Medicine and Health Sciences, Integrated Personalized and Precision Oncology Network (IPPON), University of Antwerp, Antwerp, Belgium; 4 Departments of Otolaryngology-Head and Neck Surgery, and Radiology & Nuclear Medicine, Cancer Center Amsterdam, Amsterdam UMC, Vrije Universiteit Amsterdam, Amsterdam, the Netherlands; 5 Department of Radiotherapy, AZ Groeninge, Kortrijk, Belgium; 6 Department of Radiotherapy, AZ Turnhout, Turnhout, Belgium; 7 Department of Otolaryngology, Jessa Hospital, Hasselt, Belgium; 8 Department of Nuclear Medicine, Sint Augustinus, Wilrijk, Belgium; 9 Head and Neck Surgical Oncology, University Medical Center Utrecht, Utrecht, the Netherlands; 10 Department of Nuclear Medicine, AZ Sint Jan, Brugge, Belgium; 11 Department Otorhinolaryngology and Head & Neck Surgery, Antwerp University Hospital, Edegem, Belgium; 12 Department of Nuclear Medicine, AZ Groeninge, Kortrijk, Belgium; 13 Department of Oral and Maxillofacial Surgery, ZNA Middelheim, Antwerp, Belgium

**Keywords:** FDG-PET/CT, Locally advanced squamous cell head and neck cancer, LAHNSCC, Chemoradiotherapy, HPV

## Abstract

**Background:**

FDG-PET/CT has a high negative predictive value to detect residual nodal disease in patients with locally advanced squamous cell head and neck cancer after completing concurrent chemoradiotherapy (CCRT). However, the positive predictive value remains suboptimal due to inflammation after radiotherapy, generating unnecessary further investigations and possibly even surgery. We report the results of a preplanned secondary end point of the ECLYPS study regarding the potential advantages of dual time point FDG-PET/CT imaging (DTPI) in this setting. Standardized dedicated head and neck FDG-PET/CT images were obtained 12 weeks after CCRT at 60 and 120 min after tracer administration. We performed a semiquantitative assessment of lymph nodes, and the retention index (RI) was explored to optimize diagnostic performance. The reference standard was histology, negative FDG-PET/CT at 1 year, or > 2 years of clinical follow-up. The time-dependent area under the receiver operator characteristics (AUROC) curves was calculated.

**Results:**

In total, 102 subjects were eligible for analysis. SUV values increased in malignant nodes (median SUV_1_ = 2.6 vs. SUV_2_ = 2.7; *P* = 0.04) but not in benign nodes (median SUV_1_ = 1.8 vs. SUV_2_ = 1.7; *P* = 0.28). In benign nodes, RI was negative although highly variable (median RI = − 2.6; IQR 21.2), while in malignant nodes RI was positive (median RI = 12.3; IQR 37.2) and significantly higher (*P* = 0.018) compared to benign nodes. A combined threshold (SUV_1_ ≥ 2.2 + RI ≥ 3%) significantly reduced the amount of false-positive cases by 53% (*P* = 0.02) resulting in an increased specificity (90.8% vs. 80.5%) and PPV (52.9% vs. 37.0%), while sensitivity (60.0% vs. 66.7%) and NPV remained comparably high (92.9% vs. 93.3%). However, AUROC, as overall measure of benefit in diagnostic accuracy, did not significantly improve (*P* = 0.62). In HPV-related disease (*n* = 32), there was no significant difference between SUV_1_, SUV_2_, and RI in malignant and benign nodes, yet this subgroup was small.

**Conclusions:**

DTPI did not improve the overall diagnostic accuracy of FDG-PET/CT to detect residual disease 12 weeks after chemoradiation. Due to differences in tracer kinetics between malignant and benign nodes, DTPI improved the specificity, but at the expense of a loss in sensitivity, albeit minimal. Since false negatives at the 12 weeks PET/CT are mainly due to minimal residual disease, DTPI is not able to significantly improve sensitivity, but repeat scanning at a later time (e.g. after 12 months) could possibly solve this problem. Further study is required in HPV-associated disease.

## Background

Concurrent chemoradiotherapy (CCRT) is the standard nonsurgical approach in locoregionally advanced head and neck squamous cell carcinoma (LAHNSCC). Early detection of residual or recurrent disease may allow early intervention, usually in the form of surgical salvage [1]. Evidence that underlines the role of FDG-PET/CT in the detection of residual nodal disease after CRT with high negative predictive value (NPV) has been published [1–3]. In a recent meta-analysis by Helsen et al., the reported pooled negative predictive value (NPV) was exceptionally high (98%). However, the positive predictive value (PPV) was suboptimal at 58% (given a pre-test probability of 10%) [3]. A randomized phase III trial (PET-NECK) demonstrated a non-inferior overall survival with FDG-PET/CT-guided surveillance 12 weeks after CRT compared with planned standard neck dissection, resulting in considerably fewer operations and improving cost-effectiveness [4]. Edema, fibrosis, and inflammation after radiotherapy, however, may lead to false-positive results, generating unnecessary further investigations and surgeries [2, 5]. An interval of 10–12 weeks between the end of CRT and FDG-PET/CT scanning shows the best diagnostic performance of FDG-PET/CT [1, 2].

In the ECLYPS trial, a prospective multicenter study, we confirmed that standardized FDG-PET/CT 12 weeks after CCRT is a reliable imaging technique to rule out residual nodal disease. While overall sensitivity was lower than expected (62.5%), it was shown to be strongly time-dependent with lower detection rates for recurrences beyond 9 months after imaging [6]. Semiquantitative analysis based on an SUV threshold ≥ 2.2 resulted in a small but significant improvement in accuracy over visual assessment, although the increase in sensitivity was offset by a minor reduction in specificity [7]. These limitations could potentially be lifted by dual time point imaging (DTPI). Since FDG uptake in malignant tissue continues to increase for several hours after injection compared to an early washout in benign or inflammatory tissue [8–12], the concept of DTPI to differentiate benign from malignant tissue was first introduced in head and neck cancer in 1999 [13]. In addition, Anderson et al. provided promising data on the use of triphasic dynamic FDG-PET/CT to distinguish post-radiotherapy inflammation from malignancy [14]. By repeating FDG-PET/CT scanning at a later point in time after injection of FDG, the detection of malignancy can potentially be improved due to increased sensitivity (higher tumor-to-background ratio) and specificity (lower false-positive rate).

Moreover, the relative change in SUV over time, named the retention index (RI), has been used as a marker of malignancy. However, the optimal RI cutoff value remains to be determined [15]. To complicate matters, it has been shown that active granulomatous and infectious lesions can behave similarly to malignant lesions regarding FDG uptake even on DTPI [15]. Sathekge et al. could not differentiate malignancy from tuberculomas on DTPI, and Maturu and colleagues demonstrated an increased delayed SUV in mediastinal lymphadenopathies in sarcoidosis and tuberculosis, challenging the proposed role of dual time point FDG-PET/CT imaging in differentiating benign from malignant lesions [16, 17]. Also, regarding specificity, results can be heterogeneous due to tumor heterogeneity. Moreover, we should remain cautious drawing conclusions from the available literature since variations among studies regarding DTPI are significant as they suffer from a lack of standardization in design, variability in delay between acquisition times, and reference standards [15].

As a planned secondary endpoint in the ECLYPS trial, we investigated the added value of semiquantitative DTPI to improve the diagnostic performance of FDG-PET/CT in the detection of nodal recurrence 12 weeks after CCRT. While visual response assessment using the Hopkins criteria has demonstrated its value [18], DTPI imaging may offer additional benefit in patients with equivocal scores or when assessing head and neck-only acquisitions precluding the use of the Hopkins system. Moreover, we investigated the influence of human papillomavirus (HPV) on DTPI, since it has been reported that FDG-PET/CT may be less reliable in HPV-positive tumors [3].

## Materials and methods

### Patient population

Patients received dual time point imaging as part of the ECLYPS study protocol. The study design of this single cohort multicenter prospective trial of standardized FDG-PET/CT 12 weeks after treatment with CCRT in newly diagnosed LAHNSCC has been published previously [6]. In short, patients with LAHNSCC (clinical or radiological N2 or N3 disease, any stage and no distant metastases) were eligible. Treatment consisted of concomitant CRT. (Neoadjuvant chemotherapy was allowed.) Patients were excluded in case of a history of another malignancy or other head and neck cancer histology. Patients with a concomitant second primary tumor requiring systematic treatment were also considered ineligible. The institutional review board granted study approval, and informed consent was required for all patients.

### HPV status

Assessment of HPV status in patients with oropharyngeal squamous cell carcinoma (OPSCC) was performed by evaluation of overexpression of the surrogate marker p16 using immunohistochemistry (IHC). A positive test was confirmed by polymerase chain reaction (PCR).

### Reference standard and follow-up

The reference standard was histology by neck dissection or fine-needle aspiration cytology (FNAC). When the patient refused or if such procedure was not considered appropriate according to the local investigator, nodal involvement had to be confirmed by at least two imaging modalities. Follow-up of patients with a negative FDG-PET/CT consisted of two monthly control visits with additional imaging as deemed necessary by the clinician. All patients underwent FDG-PET/CT imaging at one year after treatment to confirm the absence of disease, unless recurrent/residual disease was histologically proven before this point. Patients refusing follow-up imaging required an additional year of negative clinical follow-up, for a total of 2 years.

### 18F-FDG-PET/CT protocol

The integrated FDG-PET/CT scan protocol that was followed in the five EARL accredited [19] centers has been described previously [7]. In short, a dedicated head and neck acquisition (arms down along the side) was performed 60 min after intravenous FDG injection combined with a low-dose CT scan for attenuation correction according to the manufacturers’ recommended settings. This acquisition was followed by a high-dose CT scan (with IV-contrast, unless contra-indicated) of the head and neck region for anatomical localization of PET-findings and diagnostic purposes. Next, a whole-body PET acquisition (vertex to mid-thigh) with low-dose CT scan used for anatomical localization and attenuation correction was performed. Lastly, sixty minutes after the first series, a second dedicated head and neck FDG-PET/CT series was acquired (120 min after FDG administration).

All patients were fasted for at least 6 h before FDG injection, and blood glucose was confirmed to be below 11 mmol/l. Sufficient hydration was given and, if possible, 20 mg of propranolol was administered 30 min before tracer injection to minimize FDG uptake by muscle tissue and brown fat. During incubation, patients were put in a comfortable position and instructed to avoid motion and unnecessary talking. The FDG dose depended on weight and scanner type but was expected to be 260–370 MBq.

Low-dose CT scans were acquired for attenuation correction, without iodinated contrast, to avoid any influence on SUV. Dedicated PET acquisitions were iteratively reconstructed using a 2–3 mm full width at half maximum (FWHM) Gaussian filter and a matrix size of at least 256 × 256 voxels. PET reconstruction was performed according to the EANM FDG-PET and PET/CT procedure [20]. Quality control of the scans included a review of the DICOM headers, uptake times, performing consistency checks, and looking for artifacts.

### Image analysis

Lymph node assessment was performed by a nuclear medicine physician with over 20 years of experience. On the dedicated head and neck acquisition at 60 min after injection, the lymph node with the highest FDG uptake was selected for each patient. As dedicated head and neck images were used, Hopkins criteria were not applicable. Consequently, selected lymph nodes were scored using a 5-point scale: (1) clearly negative, (2) probably negative, (3) equivocal, (4) probably positive, and (5) clearly positive. Quantification of selected nodes was performed by manually drawing a volume of interest (VOI) on both the 60 and 120 min acquisition, and the SUV_70_ (mean value based on 3D isocontour at 70% of the maximum pixel value) was calculated. SUV_70_ was selected since this metric proved to have the best diagnostic performance in a previous analysis of the whole-body images of the ECLYPS study [7]. When no lymph node was visible, a standard VOI (0.5 cm^3^) was placed on the location of the pretreatment lymph node with the highest FDG uptake on baseline FDG-PET/CT. The retention index (RI), a measure of relative change in SUV, was calculated in each lesion using the following formula.

$${\text{RI}} = \frac{{{\text{SUV}}_{1} - {\text{SUV}}_{2} }}{{{\text{SUV}}_{1} }} \times 100\%$$

### Statistical analysis

General measurements are reported as means with 95% confidence intervals. SUV_70_ measurements are reported as the median with interquartile range (IQR). A Mann–Whitney U test was used to compare SUV measurements in benign and malignant lymph nodes. The Wilcoxon signed ranks test was used to compare SUV measurements between the 60 and 120 min acquisition. The optimal threshold for the SUV_70_ parameter on both early and delayed images was determined using a time-dependent area under the receiver operating characteristic (AUROC) curves at a time horizon of 12 months after CCRT, under the condition to achieve the highest possible sensitivity, while specificity ≥ 80% [21, 22]. Diagnostic performance was evaluated by 2 × 2 tables, sensitivity, specificity, positive and negative predictive value, and AUROC. The predictive values of subgroup analyses were adjusted to the prevalence in the whole study population. The McNemar exact test was used to compare sensitivity and specificity. A *P* value of < 0.05 was considered to indicate a statistically significant difference. All statistical analyses were performed using SPSS version 26 (IBM, USA) and R (version 3.0.1).

## Results

In ECLYPS, 123 patients received DTPI, of which 12 patients were excluded due to protocol violations such as SUV values in the liver exceeding normal limits (*n* = 4), exceeding the time limit between scans (*n* = 5), motion artifacts (*n* = 2), or difference in time/bed position between scans (*n* = 1). Additionally, nine patients were excluded as nodal status could not be assessed: Five patients had recurrence at the primary tumor site or a distant relapse without confirmation of neck status at 12 weeks or beyond, and four patients were lost to follow-up or withdrew their informed consent, leaving 102 patients evaluable for this analysis. Patient and tumor characteristics are summarized in Table 1. The mean interval from therapy to scanning and end of follow-up was, respectively, 12.6 weeks (95% CI: 12.3–12.9) and 22.1 months (95% CI: 20.3–24.0). In this cohort, 16 patients (15.7%) had confirmed residual or recurrent nodal disease, of which 15 had residual or recurrent nodal disease within 12 months after therapy. The mean interval from scanning to detection of nodal recurrence was 113.4 days (95% CI: 54.8–172.0) Overall, the mean uptake time of the first and second dedicated head and neck acquisition was, respectively, 64.0 min (95% CI: 63.1–64.9) and 123.1 min (95% CI: 121.6–124.6), resulting in a mean additional uptake time of 59.1 min (95% CI: 57.8–60.5). The mean administered activity of FDG was 277.0 MBq (95% CI: 266.6–287.4).

**Table 1:** Patient and tumor characteristics

Characteristic	*n* = 102
*Age (years)*	
Median	59
Interquartile range	11
Gender (*n*, %)	
Male	79 (77.5)
Female	23 (22.5)
*Performance status (n, %)*	
0	83 (81.4)
1	19 (18.6)
*Tumor location (n, %)*	
Oral cavity	8 (7.8)
Nasopharynx	6 (5.9)
Oropharynx	54 (52.9)
Hypopharynx	9 (8.8)
Larynx	17 (16.7)
Occult primary	7 (6.9)
Other	1 (0.8)
*HPV status of oropharyngeal tumors (n, %)*	
Negative	21 (38.9)
Positive	32 (59.3)
NA	1 (1.8)
*Tumor differentiation status (n, %)*	
Well-differentiated	9 (8.8)
Moderately differentiated	26 (25.5)
Poorly differentiated	35 (34.3)
Undifferentiated	3 (2.9)
Not assessed	29 (28.4)
*Induction chemotherapy (n, %)*	
Yes	37 (36.3)
No	65 (63.7)
*Concomitant chemotherapy (n, %)*	
Cisplatin or carboplatin	86 (84.3)
Cetuximab w/o Gemcitabine	16 (15.7)
*Tumor stage (n, %)*	
Tx	6 (5.9)
T1 or T2	56 (54.9)
T3 or T4	40 (39.2)
*Nodal stage (n, %)*	
N2a or N2b	64 (62.7)
N2c	33 (32.4)
N3	5 (4.9)

*HPV* human papillomavirus; *N* number of subjects

### Early (SUV_1_) versus delayed (SUV_2_) SUV measurements

**Fig. 1:**
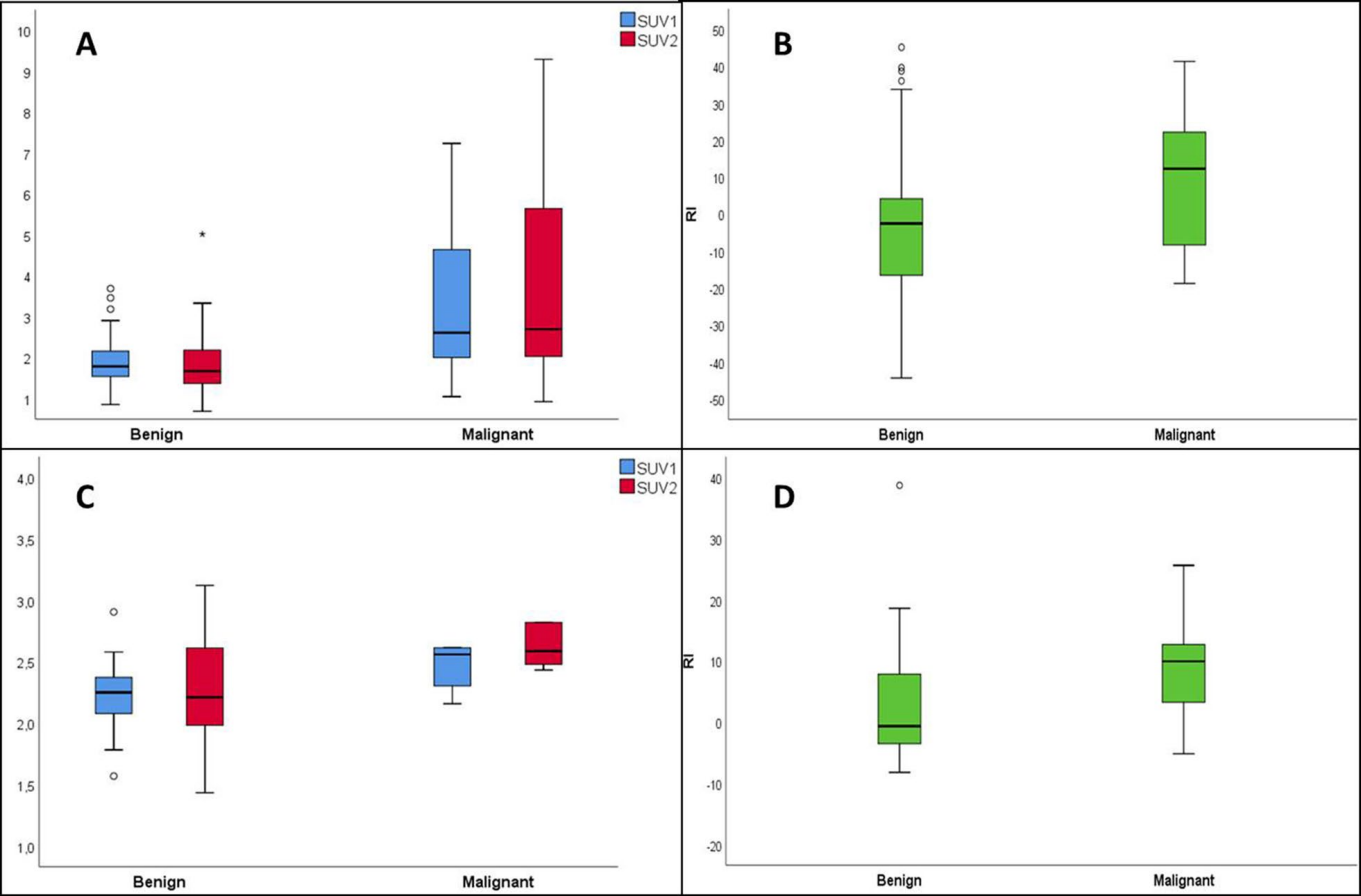
Boxplot of SUV_70_ measurements and RI of the whole study population and of visually equivocal patients. Top panel: boxplot of SUV_70_ measurements **A** and RI **B** of the whole study population (*n* = 102). Bottom panel: boxplot of SUV_70_ measurements **C** and RI **D** of visually equivocal patients (*n* = 24). The boxes represent the interquartile range, and the horizontal line represents the median. The whiskers represent the minimal (Q1 − 1.5*IQR) and maximal (Q3 + 1.5*IQR) values. The dots and asterisk indicate outliers and extreme outliers (beyond Q1 − 3*IQR or Q3 + 3*IQR), respectively. In panels B, C, and D, an extreme outlier was excluded to improve scaling

The optimal SUV cutoff to differentiate benign from malignant nodes was 2.2 for both early and late images and was independent of the chemotherapy schedule used. Both SUV_1_ and SUV_2_ were significantly higher in malignant lymph nodes compared to benign nodes (*P* = 0.01), although there was a clear overlap (Table 2, Fig. 1A). In malignant nodes, SUV_2_ was significantly higher compared to SUV_1_ (median SUV_2_ = 2.7; IQR 1.9–4.7 vs. SUV_1_ = 2.6; IQR 1.6–5.9; *P* = 0.04). In contrast, FDG uptake did not differ significantly between the delayed and early acquisition in benign nodes (median SUV_2_ = 1.7; IQR 1.5–2.2 vs. SUV_1_ = 1.8; IQR 1.4–2.2; *P* = 0.28). An SUV_1_ cutoff at ≥ 2.2 resulted in a sensitivity of 66.7% (95% CI: 38.4–88.2%), specificity of 80.5% (95% CI: 70.6–88.2%), PPV of 37.0% (95% CI: 25.2–50.7%), and NPV of 93.3% (95% CI: 87.2–96.7%), AUROC 0.74 (Table 3). The SUV_2_ threshold (optimal cutoff ≥ 2.2) resulted in similar accuracy with one fewer false-negative (FN) case at the expense of 1 additional false-positive (FP) case.

**Table 2:** Median (IQR) SUV_70_ measurements

All patients (*n* = 102)					
	Benign (*n* = 87)	Malignant (*n* = 15)	*P*_1_	*P*_2_	*P*_3_
SUV_1_	1.8 (1.5–2.2; 0.6)	2.6 (1.9–4.7; 2.8)	**0.001**		
SUV_2_	1.7 (1.4–2.2; 0.8)	2.7 (1.6–5.9; 4.3)	**0.001**	0.28	**0.04**
RI	− 2.6 (− 16.7–4.5; 21.2)	12.3 (− 11.6–25.6; 37.2)	**0.018**		

Visually equivocal patient group (*n* = 24)					
	Benign (*n* = 18)	Malignant (*n* = 6)	*P*_1_	*P*_2_	*P*_3_
SUV_1_	2.3 (2.1–2.4; 0.3)	2.6 (2.3–3.2; 0.9)	**0.04**		
SUV_2_	2.2 (2.0–2.7; 0.7)	2.6 (2.5–3.7; 1.2)	**0.03**	0.65	0.08
RI	− 0.6 (− 3.8–8.9; 12.6)	9.9 (1.2–15.9; 14.8)	0.18		

Bold was used to indicate statistical significant *P* values

*P*_1_ Mann–Whitney U test comparing SUV values of malignant and benign nodes. *P*_2_ and *P*_3_ Wilcoxon signed ranks test comparing SUV_2_ versus SUV_1_ of benign (*P*_2_) and malignant (*P*_3_) nodes

**Table 3:** Diagnostic performance of SUV_70_ measurements regarding nodal recurrence within 12 months after CCRT

	AUROC (95% CI)	TN	FN	TP	FP	Sensitivity (95% CI)	Specificity (95% CI)	PPV (95% CI)	NPV (95% CI)	*P*
*All patients (n = 102)*										
SUV_1_ (≥ 2.2)	0.74 (0.59–0.88)	70	5	10	17	66.7% (38.4–88.2%)	80.5% (70.6–88.2%)	37.0% (25.2–50.7%)	93.3% (87.2–96.7%)	
SUV_2_ (≥ 2.2)	0.76 (0.62–0.90)	69	4	11	18	73.3% (44.9–92.2%)	79.3% (69.3–87.3%)	37.9% (26.8–50.5%)	94.4% (87.8–97.5%)	0.58
SUV_1_ + RI (≥ 3%)	0.75 (0.60–0.91)	79	6	9	8	60.0% (32.3–83.7%)	90.8% (82.7–96.0%)	52.9% (34.1–71.0%)	92.9% (87.6–96.1%)	0.62
*Equivocal (n = 24)*										
SUV_1_ (≥ 2.2)	0.67 (0.39–0.89)	9	1	5	9	83.3% (35.9–99.6%)	50.0% (26.0–74.0%)	22.3% (13.8–34.0%)	94.6% (73.3–99.1%)	
SUV_2_ (≥ 2.2)	0.78 (0.56–0.94)	10	0	6	8	100% (54.1–100%)	55.6% (30.8–78.5%)	27.9% (18.8–39.4%)	100%(65.5–100%)	0.29
SUV_1_ + RI (≥ 3%)	0.72 (0.47–0.97)	14	2	4	4	66.7% (22.3–95.7%)	77.8% (52.4–96.6%)	34.1% (15.5–59.2%)	93.1% (81.0–97.7%)	0.58
*HPV-negative OPSCC (n = 21)*										
SUV_1_ (≥ 2.2)	0.82 (0.60–1)	12	1	5	3	83.3% (35.9–99.6%)	80.0% (51.9–95.7%)	41.8% (19.7–67.8%)	96.5% (82.1–99.4%)	
SUV_2_ (≥ 2.2)	0.88 (0.69–1)	14	1	5	1	83.3% (35.9–99.6%)	93.3% (68.1–99.8%)	68.3% (23.9–93.7%)	97.0% (84.4–99.5%)	0.14
SUV_1_ + RI (≥ 3%)	0.88 (0.69–1)	14	1	5	1	83.3% (35.9–99.6%)	93.3% (68.1–99.8%)	68.3% (23.9–93.7%)	97.0% (84.4–99.5%)	0.14
*HPV-associated OPSCC (n = 32)*										
SUV_1_ (≥ 2.2)	0.56 (0.20–0.93)	23	2	1	6	33.3% (0.8–90.6%)	79.3% (60.3–92.0%)	21.7% (4.6–61.6%)	87.4% (75.2–94.0%)	
SUV_2_ (≥ 2.2)	0.75 (0.42–1)	24	1	2	5	66.7% (9.4–99.2%)	82.8% (64.2–94.2%)	40.0% (17.7–67.3%)	93.5% (74.3–98.6%)	0.29
SUV_1_ + RI (≥ 3%)	0.47 (0.14–0.80)	27	3	0	2	0.0% (0.0–70.8%)	93.1% (77.2–99.2%)	0.0%(0.0–80.2%)	84.4% (83.0–85.6%)	0.57

*P* comparison of AUROC values versus the SUV_1_ metric

### The retention index

In benign lymph nodes, median RI was negative although highly variable (median RI = − 2.6; IQR − 16.7–4.5; 21.2), while in malignant nodes median RI was positive (median RI = 12.3; IQR − 11.6–25.6; 37.2) and significantly higher (*P* = 0.018) compared to benign nodes, although there was a clear overlap (Table 2, Fig. 1B). Exploration of potential RI cutoffs, when used in combination with the SUV_1_ threshold (≥ 2.2), yielded optimal results at RI ≥ 3%. This combined threshold (SUV_1_ + RI) significantly reduced FP cases by 53% (*n* = 9) at the expense of increasing FN cases by 20% (*n* = 1) compared to the SUV_1_ threshold alone (McNemar exact *P* = 0.02). This combination consequently leads to a marked increase in specificity (90.8% vs. 80.5%, + 10.3%) and PPV (52.9% vs. 37.0%, + 15.9%), while NPV remained comparably high (92.9% vs. 93.3%, − 0.4%) (Table 3). However, the difference in AUROC, as overall measure of benefit in diagnostic accuracy, was not significant (*P* = 0.62).

### The “visually equivocal” cohort

Visual assessment of the most intense nodal lesion on the early scan assigned a score of 1 to 72 LNs (70.6%), score 2 to 11 LNs (10.8%), score 3 to 10 LNs (9.8%), score 4 to 3 LNs (2.9%), and score 5 to 6 LNs (5.9%). Excluding patients with either a score of 1 or 5 (clear negative and positive cases, respectively) resulted in a cohort of 24 equivocal cases, of which 6 (25%) patients had residual or recurrent lymph node disease within 12 months after the end of chemoradiation. In this subgroup, 10 patients (41.7%) had HPV-associated OPSCC and 4 patients (16.7%) had HPV-negative OPSCC. On both the early and the delayed acquisition, SUV was significantly higher in malignant nodes compared to benign nodes (Table 2). However, neither malignant nor benign nodes showed significant changes in FDG uptake over time (Table 2, Fig. 1C). Consequently, RI was not significantly higher in malignant nodes compared to benign nodes (*P* = 0.2) (Table 2, Fig. 1D). Applying the optimal SUV threshold on the early acquisition (SUV_1_ ≥ 2.2) resulted in a sensitivity of 83.3% (95% CI: 35.9–99.6%), specificity of 50.0% (95% CI: 26.0–74.0%), PPV of 22.3% (95% CI: 13.8–34.0%), and NPV of 94.6% (95% CI: 73.3–99.1%) with an AUROC of 0.67 (95% CI: 0.39–0.89). The same threshold on the delayed acquisition reduced FN and FP cases by *n* = 1 (AUROC = 0.78), leading to an improved sensitivity (100% vs. 83.3%) and specificity (55.6% vs. 50.0%) as compared to the early time point. However, the difference in AUROC was not statistically significant (*P* = 0.29). Combining the SUV_1_ threshold with an RI cutoff at 3% yielded an increase in specificity (77.8% vs. 50.0%, + 27.8%) by reducing FP cases (*n* = 5) at the cost of 1 additional FN case (sensitivity of 66.7% vs. 83.3%; − 16.6%) (Table 3).

### Impact of human papillomavirus

**Fig. 2:**
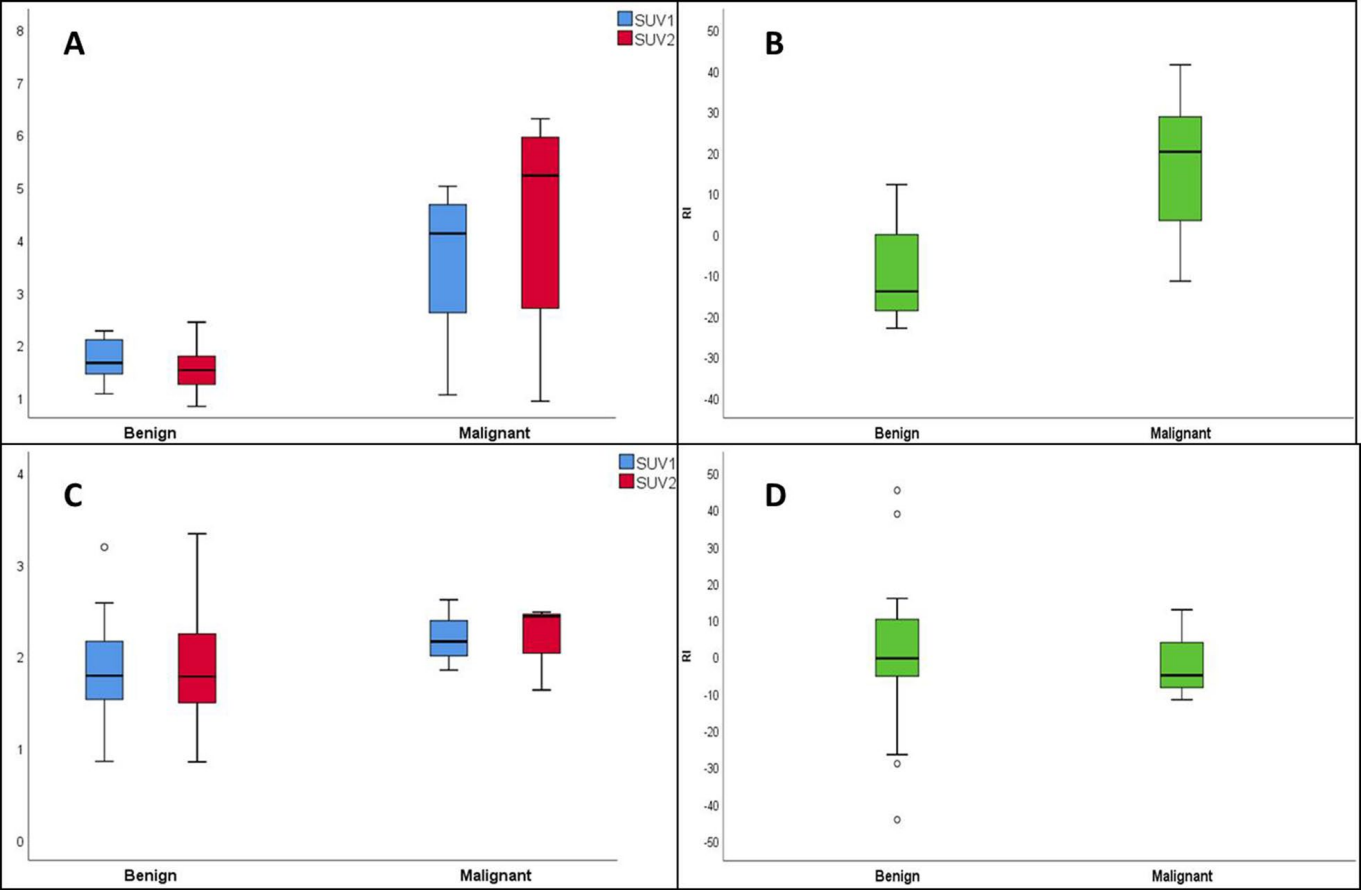
Boxplot of SUV_70_ measurements and RI in HPV-negative and HPV-associated OPSCC. Top panel: Boxplot of SUV_70_ measurements **A** and RI **B** in HPV-negative OPSCC. Bottom panel: Boxplots of SUV_70_ measurements **C** and RI **D** of HPV-associated OPSCC. The boxes represent the interquartile range, and the horizontal line represents the median. The whiskers represent the minimal (Q1 − 1.5*IQR) and maximal (Q3 + 1.5*IQR) values. The dots represent outliers. In panel D, two extreme outliers were excluded to improve scaling

Out of the 102 patients in our study, 54 patients had oropharyngeal squamous cell cancer (OPSCC), of which 32 patients had HPV-associated OPSCC, 21 patients were HPV-negative, and in one patient HPV status was not assessed (Table 1). In lymph nodes of patients with HPV-negative OPSCC, SUV_1_ and SUV_2_ were significantly higher in malignant nodes compared to benign nodes, whereas in nodes of HPV-associated OPSCC, SUV was not significantly different (Table 4, Fig. 2). Moreover, in nodes of HPV-negative OPSCC, delayed imaging revealed a significant decrease in SUV in benign nodes (*P* = 0.02) and a borderline significant increase in SUV in malignant nodes (*P* = 0.07). In contrast, nodes of HPV-associated OPSCC patients had no significant change in SUV over time in neither benign nor malignant nodes (Table 4, Fig. 2), although the small amount of malignant nodes after treatment (*n* = 3) in HPV-associated disease precludes any firm conclusions.

**Table 4:** Median (IQR) SUV_70_ measurements in OPSCC stratified by HPV status

HPV-negative OPSCC (*n* = 21)					
	Benign (*n* = 15)	Malignant (*n* = 6)	*P*_1_	*P*_2_	*P*_3_
SUV_1_	1.7 (1.3–2.1; 0.8)	4.1 (2.2–4.7; 2.5)	**0.02**		
SUV_2_	1.5 (1.2–1.8; 0.6)	5.2 (2.2–6.0; 3.8)	**0.016**	**0.02**	0.07
RI	− 14.1 (− 18.9–0.0; 18.9)	20.0 (− 0.4–31.8; 32.2)	**0.006**		

HPV-associated OPSCC (*n* = 32)					
	Benign (*n* = 29)	Malignant (*n* = 3)	*P*_1_	*P*_2_	*P*_3_
SUV_1_	1.8 (1.5–2.2; 0.7)	2.2^a^	0.17		
SUV_2_	1.8 (1.5–2.3; 0.8)	2.4^a^	0.26	0.93	1
RI	− 0.5 ( − 5.5–11.8; 17.3)	− 0.5^a^	0.67		

Bold was used to indicate statistical significant *P* values

*P*_1_: Mann–Whitney U test comparing benign versus malignant nodes. *P*_2_, *P*_3_: Wilcoxon signed ranks test comparing SUV_2_ versus SUV_1_ in benign (*P*_2_) and malignant (*P*_3_) nodes

^a^IQR could not be calculated

In HPV-negative OPSCC, the optimal threshold of the SUV_1_ parameter (≥ 2.2) resulted in a sensitivity of 83.3% (95% CI: 35.9–99.6%), specificity of 80.0% (95% CI: 51.9–95.7%), PPV of 41.8% (95% CI: 19.7–67.8%), and a NPV of 96.5% (95% CI: 82.1–99.4%), with an AUROC of 0.82. On the delayed acquisition, specificity increased to 93.3% (+ 13.3%), while sensitivity was preserved. This corresponded to a marked increase (+ 26.5%) in PPV (68.3%, 95% CI: 23.9–93.7%), while NPV remained comparably high (97.0%; 95% CI: 84.4–99.5) (Table 3). Analogous to our previous analyses, an optimal RI cutoff was explored in combination with the SUV_1_ parameter revealing an optimal cutoff at 3%. However, the diagnostic accuracy of the combined threshold was identical to the SUV_2_ (≥ 2.2) parameter on the delayed acquisition. There was no statistically significant difference in AUROC values. In patients with HPV-associated OPSCC, the diagnostic performance of the SUV_1_ parameter suffered from low sensitivity (33.3%; 95% CI: 0.8–90.6%). The SUV_2_ threshold improved the sensitivity, while specificity remained comparable. A combination of the SUV_1_ threshold with an RI cutoff yielded no benefit in diagnostic performance as it improved specificity at an unacceptable reduction in sensitivity (Table 3).

## Discussion

As a part of the ECLYPS trial, we investigated the potential of dual time point imaging to improve the diagnostic accuracy of FDG-PET/CT for the detection of residual nodal disease in LAHNSCC 12 weeks after CRT [1, 2].

Overall, FDG uptake in malignant nodes was significantly higher compared to benign nodes on both early and delayed images. Moreover, FDG kinetics was different with a significantly increased uptake over time in malignant nodes, while in benign nodes the FDG uptake remained stable. Therefore, the retention index was significantly higher in malignant nodes. In the subgroup of HPV + OPSCC patients, different FDG kinetics were observed, even though the low number of true positive lymph nodes in this cohort hampers the interpretation. First, SUV in HPV + malignant nodes was lower compared to those of HPV-negative OPSCC and no longer significantly different from the SUV in benign nodes. Also, the RI between malignant and benign nodes was not significantly different in HPV-associated OPSCC. These observations add to the increasing evidence that the different biology of HPV-associated OPSCC may affect the optimal timing and interpretation of FDG-PET/CT in this setting [3].

While our study confirmed the different FDG kinetics between benign and malignant nodes, the added value of DTPI to improve the diagnostic performance of FDG-PET/CT to detect residual nodal disease was limited since AUROC values for the DTPI metrics (SUV_2_ and SUV_1_ combined with RI ≥ 3%) were similar compared to SUV_1_ alone.

The combination of the SUV_1_ threshold with an RI cutoff at 3% yielded an increase in specificity at the expense of a minor reduction in sensitivity. This is in line with the review on DTPI by Cheng et al., who observed that delayed imaging, when applying the same criterion to define malignancy as on early imaging, will increase sensitivity at the cost of specificity. In contrast, the use of a stricter criterion (i.e., SUV + RI) may increase specificity, sometimes at the expense of sensitivity [15].

**Fig. 3:**
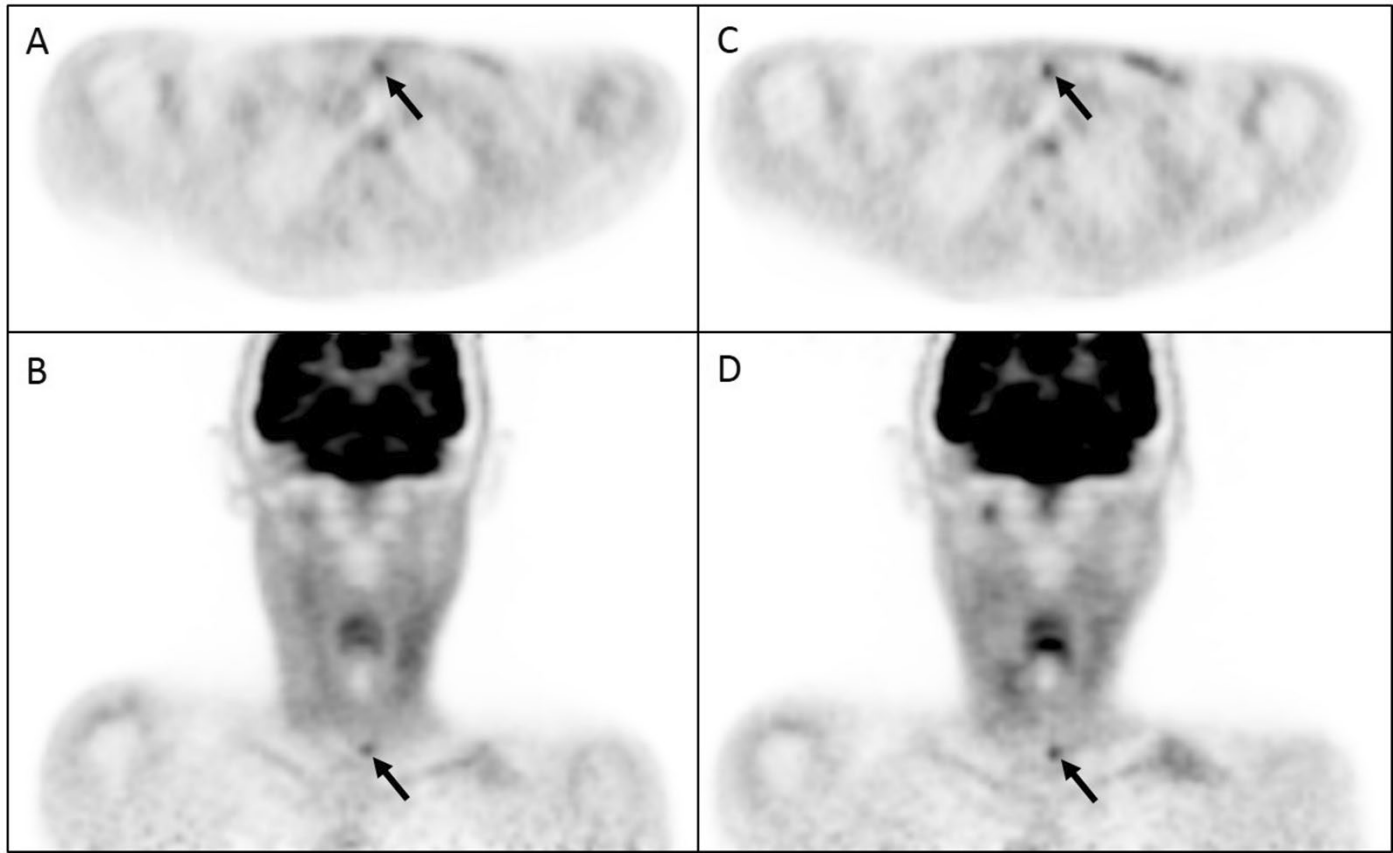
PET images of a patient with T2N2cM0 OPSCC. Early axial and coronal **A**, **B** and delayed **C,** **D** PET images of a patient with T2N2cM0 OPSCC. A suprasternal lymph node with an equivocal visual score and a SUV under the threshold (SUV_1_ = 2.1) was identified on the early head and neck PET acquisition 60 min after FDG administration (black arrow; **A**, **B**). On the delayed PET acquisition, this lymph node remained visually equivocal (black arrow; **C**, **D**). However, the SUV increased above the threshold (SUV_2_ = 2.4; RI = 12.7%). Recurrence was histologically proven by neck dissection

Delayed imaging had a limited effect on sensitivity since only 1 of the 5 false-negative nodes on early imaging showed FDG uptake above the threshold on the late scan (Fig. 3). This node had an equivocal visual score both on early and delayed images and a positive RI (RI = 12.7%). In contrast, the remaining 4 false-negative cases had a visual score of 1 on both time point images and a negative RI. Furthermore, the time interval between FDG-PET/CT and the clinical detection of recurrence was longer in these 4 cases (between 227 and 283 days) compared to only 27 days for the node that was detected only on the delayed acquisition. Since 4 out of 5 false-negative cases on the PET/CT at 12 weeks after CCRT were detected as subclinical but FDG-avid disease on the routinely acquired PET/CT scan 1 year after chemoradiation, we postulate that the false-negative results of FDG-PET/CT at 12 weeks after CCRT were due to either minimal residual disease still below the detection limit of the PET camera or due to early tumor recurrence, not yet present at 12 weeks after CCRT. This finding indicates that not DTPI, but repeat scanning at a later time interval after therapy (for instance, 1 year after CCRT) is required.

Regarding specificity, the combination of the SUV_1_ and RI threshold significantly reduced the amount of false-positive cases (*n* = 9; − 53%) at the expense of 1 additional FN case. This increase in specificity was not observed using only the SUV_2_ parameter. Based on the analysis of the OPSCC subgroup, the effect of an increased specificity by adding the RI threshold might be tumor-specific, as in HPV-negative OPSCC it increased specificity (reduced FP cases by *n* = 2) while preserving sensitivity. While in HPV-associated OPSCC increased specificity came at an unacceptable offset in sensitivity. However, as mentioned previously, these findings are prone to bias due to the low amount of malignant nodes in these subgroups, and therefore, we should refrain from drawing definitive conclusions.

In our study, we performed delayed time point imaging at 2 h after injection of FDG, as is the case in most DTPI studies [15]. However, there is no consensus on an optimal delay time for either the initial or delayed time point of image acquisition leading to considerable variability in DTPI studies. Cheng et al. proposed a delay time for DTPI of at least 2 h and ideally even 3 h after FDG injection [15]. In further research on DTPI, a standardized approach toward delayed image acquisition times should be considered.

Finally, we investigated the potential benefit of DTPI in patients with an equivocal visual read for residual neck disease. Standardized qualitative interpretation criteria for response assessment in head and neck cancer are commonly used in clinical practice. In our study, we used dedicated head and neck PET acquisitions both at 60 and 120 min and therefore an adapted 5-point scoring system was used instead of the Hopkins criteria. In this subgroup of 24 patients, SUV values did not alter significantly over time, neither in benign nor malignant nodes. Accordingly, RI was not significantly different, and SUV_1_ combined with RI did not improve accuracy. These disappointing results could in part be explained by the fact that almost half (41.7%) of the patients in this equivocal group had HPV-associated OPSCC, where the advantage of DTPI is uncertain.

## Conclusion

DTPI did not improve the overall diagnostic accuracy of FDG-PET/CT to detect residual disease 12 weeks after chemoradiation. Due to differences in tracer kinetics between malignant and benign nodes, DTPI improved the specificity, but at the expense of a loss in sensitivity, albeit minimal. Hence, the added value in routine practice will be marginal, since clinicians will opt for histological confirmation of FDG-avid lymph nodes, even if kinetics suggest benign disease, in order to not deny a patient potential curative salvage surgery.

In HPV-associated OPSCC, FDG uptake and tracer kinetics were not significantly different between benign and malignant nodes, probably reflecting the difference in underlying biology in this subgroup. Evaluation in larger patient groups is needed to define the optimal imaging strategy in this subgroup.

False negatives were often associated with late clinical relapses, probably due to minimal residual disease or early recurrence not yet present at the 12 weeks PET/CT scan. Repeat scanning at a later time point during follow-up (e.g., 12 months after chemoradiation) could help to detect these recurrences early, although progressive fibrosis and scarring after irradiation may complicate subsequent salvage surgery at later time points.

## Data Availability

The datasets used and/or analyzed during the current study are available from the corresponding author on reasonable request.

## Abbreviations

AUROC: Area under the receiver operator characteristics
CI: Confidence interval
CRT: Chemoradiotherapy
DICOM: Digital Imaging and Communications in Medicine
DTPI: Dual time point imaging
EANM: European Association of Nuclear Medicine
FDG: Fluorodeoxyglucose
FN: False negative
FNAC: Fine needle aspiration cytology
FP: False positive
HPV: Human papillomavirus
IHC: Immunohistochemistry
IV: Intravenous
LAHNSCC: Locally advanced head and neck squamous cell cancer
NPV: Negative predictive value
OPSCC: Oropharyngeal squamous cell cancer
PCR: Polymerase chain reaction
PET/CT: Positron emission tomography/computed tomography
PPV: Positive predictive value
RI: Retention index
SUV: Standard uptake value
VOI: Volume of interest
